# Characteristics of Pachychoroid Diseases and Age-Related Macular Degeneration: Multimodal Imaging and Genetic Backgrounds

**DOI:** 10.3390/jcm9072034

**Published:** 2020-06-29

**Authors:** Kenji Yamashiro, Yoshikatsu Hosoda, Masahiro Miyake, Sotaro Ooto, Akitaka Tsujikawa

**Affiliations:** 1 Department of Ophthalmology and Visual Sciences, Kyoto University Graduate School of Medicine, Kyoto 606-8507, Japan; kubrick@kuhp.kyoto-u.ac.jp (Y.H.); miyakem@kuhp.kyoto-u.ac.jp (M.M.); ohoto@kuhp.kyoto-u.ac.jp (S.O.); tujikawa@kuhp.kyoto-u.ac.jp (A.T.); 2 Department of Ophthalmology, Japanese Red Cross Otsu Hospital, Otsu 520-8511, Japan

**Keywords:** age-related macular degeneration, genetic association, indocyanine green angiography, optical coherence tomography, pachychoroid disease

## Abstract

The emergence of pachychoroid disease is changing the concept of age-related macular degeneration (AMD). The concept of pachychoroid diseases was developed through clinical observation of multimodal images of eyes with AMD and central serous chorioretinopathy; however, recent genetic studies have provided a proof of concept for pachychoroid spectrum disease, which should be differentiated from drusen-driven AMD. The genetic confirmation of pachychoroid concept further provides novel viewpoints to decode previously reported findings, which facilitates an understanding of the true nature of pachychoroid diseases and AMD. The purpose of this review was to elucidate the relationship between pachychoroid diseases and AMD by interpreting previous findings on pachychoroid diseases and AMD from the novel viewpoints of genetic associations. We confirmed that previous genetic studies supported the concept of pachychoroid diseases. From a genetic viewpoint, the presence of thick choroid and the presence of choroidal vascular hyperpermeability were important characteristics of pachychoroid spectrum diseases. Previous studies have also suggested the classification of polypoidal choroidal vasculopathy (PCV) into two subtypes, pachychoroid neovasculopathy and drusen-driven PCV. Genetic viewpoints will be beneficial to rearrange subtypes of drusen-driven AMD and pachychoroid spectrum diseases. Further genetic studies are needed to investigate pachyvessels, pachydrusen and the significance of polypoidal lesions in pachychoroid neovasculopathy and drusen-driven AMD/PCV.

## 1. Introduction

The concept of pachychoroid spectrum diseases has been established within the past 10 years [1,2,3] and has been changing the diagnosis and concept of age-related macular degeneration (AMD). Pachychoroid diseases develop in eyes with thick choroid in which pachychoroid induces various diseases such as central serous chorioretinopathy (CSC), pachychoroid pigment epitheliopathy, pachychoroid neovasculopathy, and pachychoroid geographic atrophy (GA). Although the choroid is usually thicker in pachychoroid diseases than in AMD, pachychoroid diseases share many clinical manifestations with AMD. When we re-examined Japanese neovascular AMD patients who were diagnosed according to the criteria used before the introduction of the pachychoroid concept, we found that at least 20% of AMD patients had to be diagnosed as eyes with choroidal neovascularization (CNV) that was secondary to pachychoroid or pachychoroid neovasculopathy [4]. Furthermore, our recent study using machine learning suggested that 46% of previously diagnosed AMD in Japanese patients had to be diagnosed as pachychoroid neovasculopathy [5].

Retina specialists have accepted the concept of pachychoroid spectrum disease since its proposal. However, a clear definition differentiating pachychoroid neovasculopathy from neovascular AMD has not been established. Optical coherence tomography (OCT) and indocyanine green angiography (ICGA) can demonstrate the characteristic findings of pachychoroid neovasculopathy, such as a thick choroid, dilated choroidal vessels, and choroidal vascular hyperpermeability. However, such characteristics are observed not only in eyes with pachychoroid neovasculopathy but also in eyes with neovascular AMD. Furthermore, there had been no pathophysiologically rational evidence for the significance of differentiating pachychoroid neovasculopathy from neovascular AMD. Definitive differences in treatment responses to anti-VEGF treatment have not been reported between these two diseases. It had not been clearly elucidated whether thick choroid development and the ensuing CNV in the pachychoroid neovasculopathy shares common backgrounds with the development of neovascular AMD or whether their backgrounds differ.

**Figure 1:**
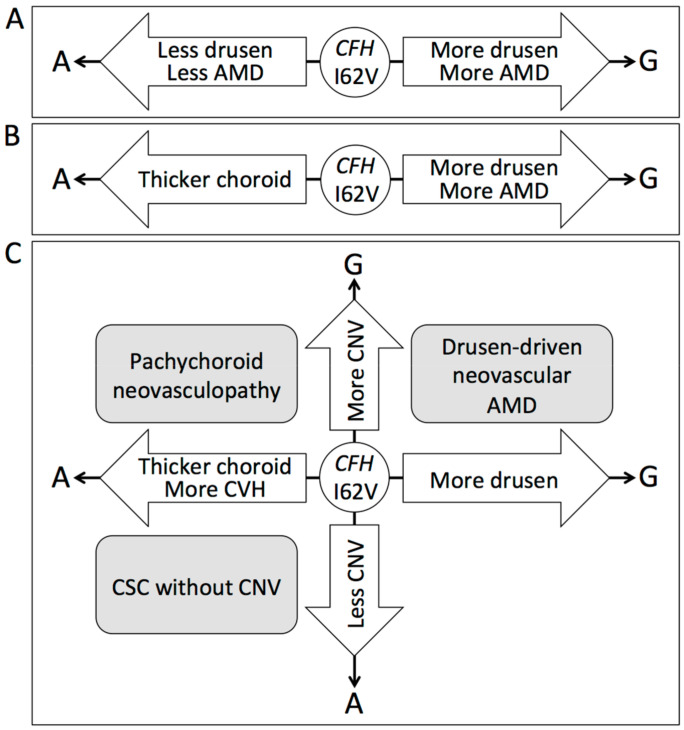
Association of *CFH* gene polymorphism with age-related macular degeneration (AMD) and pachychoroid. A single nucleotide polymorphism of I62V (rs800292) in *CFH* has a significant association with the development of AMD. G allele is a risk allele for drusen and AMD, while A allele is a protective allele for drusen and AMD (**A**). I62V in *CFH* has opposite effects on AMD and pachychoroid. G allele is a risk allele for drusen and AMD but a protective allele for pachychoroid (**B**). I62V in *CFH* has similar effects to choroidal neovascularization (CNV) development in AMD and pachychoroid diseases. G allele is a risk allele for CNV development both in pachychoroid diseases and drusen-driven AMD (**C**).

Recently, we have discovered a key gene, *CFH*, that shows that pachychoroid diseases and AMD belong to opposite disease spectrums [6]. Although *CFH* is an established gene that is associated with development of the drusen and AMD (Figure 1A), *CFH* risk alleles for the development of drusen and AMD protect against pachychoroid development, while the *CFH* risk alleles for pachychoroid protect against drusen and AMD development (Figure 1B). This finding provided a proof of concept for pachychoroid disease to be considered a distinct clinical entity that should be differentiated from drusen-driven AMD. Furthermore, our recent study revealed that the *CFH* and *ARMS2* genes determine the fate of eyes with CSC. CSC in a patient with the protective allele of *CFH* against AMD tends to resolve earlier without treatment; however, in patients with the risk alleles of *CFH* and *ARMS2* for AMD, CSC tends to progress to pachychoroid neovasculopathy through development of CNV even after the resolution of serous retinal detachment due to CSC [7]. In contrast, *VIPR2* promotes pachychoroid and CSC development but does not affect the progression to pachychoroid neovasculopathy. It would be suggested that the protective alleles of *CFH* against AMD promote pachychoroid and subsequent CSC development but promote early resolution of CSC and suppress the progression from CSC to pachychoroid neovasculopathy, while the risk alleles of *CFH* for AMD promote the progression of pachychoroid neovasculopathy from *VIPR2*-induced CSC. Before the pachychoroid era, many genetic studies had evaluated the characteristics of AMD and CSC, although AMD and CSC are multifactorial diseases caused by both genetic and environmental factors. After the genetic confirmation of pachychoroid concept, it is necessary to review the characteristic findings in OCT and ICGA from the viewpoint of genetic associations to help us precisely understand pachychoroid diseases and AMD.

## 2. Characteristic Findings in Imaging Analyses for Neovascular AMD

Neovascular AMD is usually classified into the following three subtypes: retinal angiomatous proliferation (RAP), polypoidal choroidal vasculopathy (PCV), and typical neovascular AMD (tnAMD). In a clinical setting, these subtypes are differentiated with the help of fundus imaging analyses such as OCT and ICGA.

The RAP lesion has been recognized in advanced AMD for almost 30 years. In 1992, Hartnett et al. classified fluorescein angiography (FA) images of pigment epithelium detachment (PED) associated with drusen [8]. A proposed PED type was “retinal vascular abnormality” type that was characterized by an “outer retinal angiomatous lesion” in the center of the PED. In 1996, they further analyzed ICGA images of the retinal angiomatous lesion and named it as “retinal vascular anomalous complex (RVAC)” [9]. In 1995, a French study group demonstrated that the vascular lesion of RVAC was a “chorioretinal anastomosis (CRA)” using ICGA analysis [10]. In 2001, Gass presented a hypothesis at the Vitreous Society Meeting that small foci of superficial retinal or preretinal blood was an important clinical sign of occult-CRA (OCRA) development and later his group described an OCRA hypothesis using five stages of OCRA development, where the OCRA lesion starts to develop within the choroid [11]. This hypothesis was confirmed using prospective OCT examination by another group [12]. In 2001, Yannuzzi et al. proposed to term RVAC/OCRA as “RAP” by applying a three-stage classifying system, where the RAP lesion starts to develop within the retina [13]. After a decade of intensive investigation, the vascular lesion of RVAC/OCRA/RAP was found to originate both from the retinal vessels and choroidal vessels, and a new term of “type 3 neovascularization” was proposed for this entity [14].

PCV was first presented by Yannuzzi at the Macular Society Meeting in 1982 as “Choroidal Polypoid Vasculopathy” and his group named this disease “Idiopathic Polypoidal Choroidal Vasculopathy” in 1990 [15]. During nearly a decade of investigations on idiopathic polypoidal choroidal vasculopathy, its clinical spectrum has been expanded [16], and the term “polypoidal choroidal vasculopathy (PCV)” has become widely used [17]. The early report on idiopathic polypoidal choroidal vasculopathy did not clearly mention whether the vascular lesion was a CNV or a choroidal vascular abnormality [15]. However, detailed ICGA evaluations suggested that the vascular lesion of PCV was a CNV [18], and the term “polypoidal CNV” has become widely used for PCV vascular lesions [17,19,20,21]. Since around 2000, many studies have regarded PCV as a form of type 1 CNV that locates beneath the retinal pigment epithelium (RPE) layer [20,22,23].

Although PCV was initially regarded as a distinct clinical entity that could be differentiated from AMD or other macular diseases with CNV [16], Yannuzzi and colleagues described in 2004 that “PCV represents a subtype of CNV in AMD” [24]. After the inclusion of PCV into AMD subtypes, the prevalence of PCV among neovascular AMD was extensively studied in Asia where ICGA was widely performed for macular degenerative diseases. Among neovascular AMD in Asians, 22–62% has been reported to be PCV [21,25,26,27,28,29,30,31], while the rate of PCV is 8–9% in Caucasian cases of neovascular AMD [17,31].

When the ICGA examination was introduced, it was proposed that ICGA could differentiate PCV from tnAMD by detecting branching vascular networks and polypoidal lesions [18]. OCT is also useful for detecting polypoidal lesions as anteriorly protruding lesions and branching vascular networks as double-layer signs [32,33]. However, as more clinical studies on PCV have been conducted, particularly in Asia, more retina specialists have realized the difficulty in differentiating tnAMD and PCV, and it has been suspected that PCV is not a distinct clinical entity among neovascular AMD.

Polypoidal lesions are observed as hot spots with ICGA. However, hot spots are not always polypoidal lesions. A hot spot is also a characteristic finding for RAP [13]. A study on 220 eyes with neovascular AMD in the US reported that 62% of the hot spots were observed in PCV, 30% were observed in RAP, and 8% were observed in tnAMD with type 1 CNV [23]. Hot spots can also be observed in tnAMD. A study from Belgium reported that hot spots were observed in 39% of patients with tnAMD with type 1 CNV [22].

OCT is also useful to detect polypoidal lesions [32,34,35]; however, a quantitative definition for polypoidal lesions has not yet been established. When the lesion is small but of a slightly detectable size, it is difficult to diagnose whether it is a polyp or just a small RPE elevation on the verge of type 1 CNV. If the lesion is identified as a polyp, it is diagnosed as PCV, while if it is judged not to be a polyp, it is diagnosed as tnAMD with type 1 CNV.

As the branching vascular network and polypoidal lesion in PCV usually locate beneath the RPE as type 1 CNV, vascular lesion in PCV is easily differentiated from type 2 CNV in tnAMD that locates above the RPE. However, it is not always easy to differentiate the branching vascular network in PCV from type 1 CNV in tnAMD even with ICGA [21]. It had been proposed that the branching vascular network could be differentiated from CNV of tnAMD by observing the disappearance of the dye in the very late phase of ICGA because the branching vascular network did not show diffuse late staining that is observed in CNV of tnAMD as a plaque [16]. However, later studies have shown that the branching vascular network can be detected as a plaque in late ICGA [22,35,36,37]. In OCT images, the branching vascular network is observed as double-layer sign [33]. However, type 1 CNV was also observed as a double layer. A study from China reported that the double-layer sign was observed in 92% of PCV and 28% of tnAMD [38].

As polypoidal lesions spontaneously resolve or regress with treatment, eyes with PCV can sometimes show only type 1 CNV without polypoidal lesions, which cannot be differentiated from tnAMD with type 1 CNV. In contrast, in Japanese patients, it has been demonstrated that 28% of eyes with tnAMD develop polypoidal lesions during the follow-up period of two years [39].

## 3. Characteristic Findings in Imaging Analyses for CSC

Although the pathogenesis of CSC has not been clearly elucidated, detailed analyses of ICGA have revealed that choroidal vascular hyperpermeability is a characteristic finding of CSC, and it is observed in almost all eyes with CSC [40,41,42,43]. Therefore, it has been thought that choroidal vascular hyperpermeability is the principal underlying pathophysiologic abnormality of CSC. Thicker choroid is another recently discovered characteristic of CSC. After the introduction of swept source OCT or enhanced depth imaging techniques using spectral domain OCT, choroidal thickness has become a measurable index for studies on macular diseases. The enhanced depth imaging technique was first used for highly myopic eyes in 2009 [44]. Soon after this application, the same group reported that the mean subfoveal choroidal thickness (SFCT) was measured as 505 ± 124 µm in 28 eyes with CSC [45]. Later studies from other groups have reported a slightly thinner average thickness of 308 to 461 µm in eyes with CSC, which was still significantly thicker than that in normal controls [46,47,48,49,50,51,52,53,54,55,56,57,58,59,60,61].

CSC is usually classified into early CSC and late CSC. The serous retinal detachment spontaneously resolves within three to six months in early CSC, while it is prolonged for more than three to six months in late CSC. In the FA examination, focal leak points are often observed in acute CSC, while diffuse leak areas are often observed in chronic CSC. Recently, chronic CSC was demonstrated as a risk factor for CNV development in eyes with CSC [62].

## 4. Comparison of Findings in Imaging Analyses between AMD and CSC

Eyes with CSC usually manifest serous retinal detachment with or without small PEDs. It is not rare for chronic CSC to develop type 1 CNV [63], and sometimes, its edge is slightly elevated as polypoidal lesions. When eyes with tnAMD/PCV develop type 1 CNV/branching vascular networks accompanied with serous retinal detachment without retinal edema, hemorrhage, fibrin exudates, or type 2 CNV, it is sometimes difficult to differentiate them from chronic CSC with type 1 CNV.

Soon after establishing the concept of PCV, ICGA was proposed to be useful in differentiating CSC with type 1 CNV from PCV by detecting branching vascular networks and polypoidal lesions [20]. However, as explained above, ICGA cannot always distinguish branching vascular networks from type 1 CNV. When the edge of the type 1 CNV is elevated in eyes with CSC, it is difficult to differentiate this finding from PCV even with OCT. Considering that PCV cannot be differentiated from tnAMD when its polypoidal lesion spontaneously resolves or regresses after treatment, ICGA and OCT also cannot always distinguish CSC with type 1 CNV from tnAMD with type 1 CNV.

Choroidal vascular hyperpermeability, a characteristic finding of CSC, was also expected to be a useful finding in ICGA for differentiating CSC from tnAMD/PCV. When choroidal vascular hyperpermeability was first examined in PCV, none of the examined 13 patients with PCV represented choroidal vascular hyperpermeability [20]. Furthermore, in a study of tnAMD with type 1 CNV, 98% of the patients did not show the transient choroidal hyperfluorescence in ICGA [43]. However, a later study from Japan found that 10% of patients with PCV exhibited choroidal vascular hyperpermeability in late ICGA [64]. Following this study, several groups further examined choroidal vascular hyperpermeability in tnAMD and PCV. Choroidal vascular hyperpermeability was reportedly observed in 27–52% of tnAMD and 31–53% of PCV in Asian patients [65,66,67,68,69,70,71,72,73]. It has also been demonstrated that 22% of eyes with choroidal vascular hyperpermeability develop type 1 CNV; 33% of which were type 1 CNV with polypoidal lesions, and 67% of them were type 1 CNV without polypoidal lesions [74]. Considering the high prevalence of choroidal vascular hyperpermeability in tnAMD/PCV, choroidal vascular hyperpermeability itself cannot be a criterion to distinguish CSC from tnAMD/PCV.

Choroidal thickness might be another useful candidate to differentiate CSC from tnAMD/PCV. The first study comparing choroidal thickness between tnAMD and PCV reported that choroidal thickness was 171.2 ± 38.5 µm in tnAMD and 438.3 ± 87.8 µm in PCV [75]. Although this drastic difference between tnAMD and PCV seemed to indicate that tnAMD and PCV could be easily differentiated by only choroidal thickness itself, later studies revealed that choroidal thickness of these two subtypes overlaps substantially but is indeed significantly thicker in PCV than in tnAMD. The mean choroidal thickness has been reported to range from 195 to 245 µm in tnAMD [47,76,77,78,79] and 243 to 320 µm in PCV [47,77,78,79,80,81]. Most studies have reported that the choroidal thickness is greater in PCV than in normal eyes and is thinner in tnAMD than in normal eyes. However, the difference in the choroidal thickness between tnAMD and PCV no longer exists when only eyes with choroidal vascular hyperpermeability are compared. The reported choroidal thickness in tnAMD with choroidal vascular hyperpermeability (278.2 ± 98.7 µm) was not significantly different from PCV with choroidal vascular hyperpermeability (283.0 ± 77.4 µm) [78].

Regarding the difference in choroidal thickness between CSC and tnAMD/PCV, many previous studies reported that choroidal thickness was greater in eyes with CSC than in eyes with PCV or tnAMD. However, the decrease in choroidal thickness with age makes comparison of choroidal thickness between CSC and tnAMD/PCV difficult. Choroidal thickness in eyes with CSC in patients older than 60 years of age was calculated to be 298.0 ± 84.0 µm [46], which was similar to those of tnAMD/PCV with choroidal vascular hyperpermeability. Furthermore, choroidal thicknesses in eyes with CSC together with focal choroidal excavation in patients older than 60 years of age were reportedly 263.5 ± 64.5 µm [53]. Choroidal thicknesses in eyes with tnAMD/PCV together with choroidal vascular hyperpermeability seem to be similar to those of eyes with CSC in patients of similar ages.

## 5. Susceptibility Genes for AMD

The first susceptibility gene for AMD, *CFH*, was discovered by the genome-wide association study (GWAS) in 2005 using Caucasian samples [82]. Later studies confirmed significant associations of *CFH* with AMD in the Asian population [83]. The complement factor H (CFH) is a key regulator of the complement system, and various components of the complement cascade have been identified in the drusen of patients with AMD [82,84,85]. Drusen is a major risk factor for developing late AMD [86,87,88], and CFH itself is found in drusen [89,90,91]. It has been also reported that *CFH* is significantly associated with both steps of drusen formation in early AMD development and the progression from early AMD to late AMD [92,93,94,95,96,97,98,99]. It should be reasonable to hypothesize that *CFH* contributes to the development of AMD through drusen-driven mechanisms.

The second susceptibility locus for AMD, *ARMS2/HTRA1*, was discovered through GWASs in 2005 and 2006 [100,101,102,103] using Caucasian samples or Asian samples. Since *ARMS2* and *HTRA1* are located within the same linkage disequilibrium block in chromosome 10, genetic studies have not clearly determined which gene is responsible for AMD development, and the term *ARMS2/HTRA1* is usually used to represent these AMD-susceptibility loci. Although the mechanisms of *ARMS2/HTRA1* to promote AMD development has not been elucidated, it has been reported that *ARMS2/HTRA1* is also significantly associated with drusen formation [95,97,98,104].

After the discovery of these two loci for AMD, many studies evaluated various candidate genes and found several susceptibility genes for AMD such as *C2/CFB*, *C3*, and *CFI*. Furthermore, later GWASs have discovered more susceptibility genes [105,106,107,108,109], and the largest meta-analysis of GWASs has confirmed that 34 loci are associated with AMD development [110]. Among them, *CFH* and *ARMS2/HTRA1* are the major two susceptibility genes for AMD both in Caucasians and Asians [109]. The associations of *CFH* and *ARMS2/HTRA1* with PCV and RAP have been confirmed in both Asians and Caucasians [83,111,112,113,114,115,116,117,118].

## 6. Genetic Associations with the Phenotype of AMD

**Figure 2:**
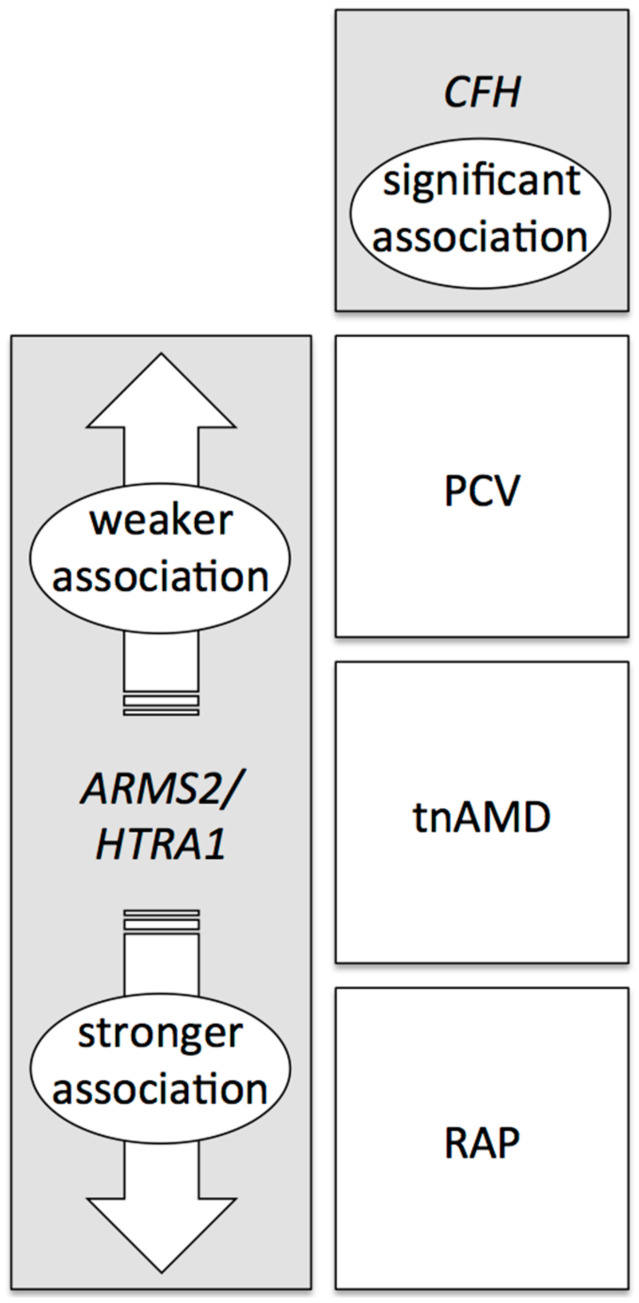
Association of *CFH* and *ARMS2/HTRA1* with subtypes of age-related macular degeneration (AMD). The association of *ARMS2/HTRA1* is weaker for polypoidal choroidal vasculopathy (PCV) and stronger for retinal angiomatous proliferation (RAP) than typical neovascular AMD (tnAMD). *CFH* does not show such significant differences in its effect size across the three neovascular AMD subtypes.

Detailed analysis revealed that the effect size of the *ARMS2/HTRA1* association was significantly different among tnAMD, PCV, and RAP in Japanese patients, while *CFH* did not show such significant differences in its effect size across the three neovascular AMD subtypes (Figure 2) [83]. The reported frequencies of the *ARMS2/HTRA1* risk allele for AMD were approximately 40% in normal controls, 55% in PCV, 65% in tnAMD, and 90% in RAP. These differences were confirmed in later studies in Asia [116,119,120]. The association of *ARMS2/HTRA1* is stronger for RAP and weaker for PCV.

Further detailed analyses revealed peculiar associations of *ARMS2/HTRA1* with PCV. Two Japanese groups and one French group tried to classify PCV into two subtypes (Table 1). One Japanese group divided PCV into smaller PCV and larger PCV, depending on the area of the vascular lesion [121]. PCVs larger than one disc area often enlarged, while PCVs smaller than one disc area rarely enlarged. The frequencies of the *ARMS2/HTRA1* risk allele for AMD were about 61% in larger PCV while 40% in smaller PCV suggesting that *ARMS2/HTRA1* was significantly associated with the development of the larger PCVs but not with the development of the smaller PCVs [121].

Another Japanese group divided PCV depending on the visibility of the feeder and draining vessels in ICGA [122,123,124]. They named lesions without visible feeder and draining vessels as “typical PCV”, assuming that such lesions represent true and genuine PCVs in a strict and narrow sense on their hypothesis that PCV must contains abnormal choroidal vessels but not CNVs. Lesions with visible feeder and draining vessels were assumed not to be true PCVs but CNV with polypoidal lesions at its edge, and this presentation was named as “polypoidal CNV”. A warning is necessary here, that the usage of “polypoidal CNV” differs from its originally used term “polypoidal CNV,” which covers all PCV as a whole, with the idea that all PCVs are CNVs and are not composed of abnormal vessels. The lesion sizes of the proposed two subtypes were approximately one disc area and 2.5 disc areas, respectively, which were similar to the reported sizes in the above study from the other Japanese group. Similar to the other Japanese group’s findings, this study also reported that the frequency of the *ARMS2/HTRA1* risk allele for AMD was significantly higher in the larger subtype but not significantly different in the smaller subtype, compared to normal controls [122].

A French group divided PCV into “idiopathic PCV” and “PCV as a subtype of neovascular AMD,” by analyzing the patient age, evolution rapidity of the vascular lesion, presence of drusen and hemorrhagic PED, and FA/ICGA and OCT images [125]. The “idiopathic PCV” had smaller lesion size, thicker choroidal thickness, and less drusen than “PCV as a subtype of neovascular AMD”. The similarity of the subtype concept and the lesion size suggests that “smaller PCV,” “typical PCV,” and “idiopathic PCV” belong to a similar subtype and “larger PCV,” “polypoidal CNV,” and “PCV as a subtype of neovascular AMD” belong to another similar subtype. *ARMS2/HTRA1* is suggested to be associated only with the larger subtypes with frequent prevalence of drusen and thinner choroid while *ARMS2/HTRA1* does not seem to be associated with the smaller subtypes with less frequent prevalence of drusen and thicker choroid.

## 7. Susceptibility Genes for CSC

Phenotypic overlap between eyes with chronic CSC and eyes with tnAMD/PCV, such as type 1 CNV, serous retinal detachment, small PEDs, and RPE disturbance has facilitated association studies of AMD-associated genes on chronic CSC. A study from the Netherlands examined 19 AMD susceptibility genes and reported that risk alleles for AMD in *CFH* worked protectively against chronic CSC development [126]. Two other studies from Japan and Greece evaluated both acute and chronic CSC together and reported similar significant associations of *CFH* with CSC [127,128]. Furthermore, recent GWAS has confirmed the genome-wide level significance of *CFH* with CSC [129].

Although these results seemed to suggest a genetic and pathophysiologic overlap between CSC and AMD, the reason the *CFH* risk alleles for AMD development protect against CSC development, while the *CFH* risk alleles for CSC protect against AMD, had not been clearly explained.

Similar to *CFH*, the *ARMS2/HTRA1* was also evaluated as a candidate gene, and one study reported that risk alleles for AMD protected against chronic CSC development in Caucasian individuals [126]. Furthermore, candidate gene studies from the US and the Netherlands have reported *CDH5*, *C4B*, and *NR3C2* as susceptibility genes for CSC [130,131,132,133]. Recent GWASs in Japan reported *SLC7A5*, *TNFRSF10A*, and *GATA5* as susceptibility genes for CSC [134,135], and recent exome sequencing studies in Caucasian identified *PIGZ*, *DUOX1*, *RSAD1*, *LAMB3*, and *PTPRB* as potential new candidate genes for chronic CSC [136,137]. However, no replication study from other groups has confirmed the association of these genes with CSC thus far.

## 8. Genetic Associations of AMD from the Viewpoint of CSC Characteristics

When eyes with tnAMD or PCV were divided into two groups according to the existence of choroidal vascular hyperpermeability, which is one of the most important characteristics for CSC, three studies from Japan have suggested that only eyes without choroidal vascular hyperpermeability show significant associations between the disease development and *CFH* or *ARMS2/HTRA1*, while eyes with choroidal vascular hyperpermeability do not show such genetic associations (Table 2 and Table 3). One report from Japan compared the frequencies of the risk alleles for AMD in *CFH* and *ARMS2/HTRA1* between PCV and normal controls and found no significant difference in the risk allele frequencies between normal control and PCV with choroidal vascular hyperpermeability, while both genes showed significantly higher risk allele frequencies in PCV without choroidal vascular hyperpermeability than in the normal control [138]. Another study from Japan evaluated both tnAMD and PCV together and also reported no significant differences in the frequencies of the risk alleles in *CFH* and *ARMS2/HTRA1* between normal controls and patients with type 1 CNV manifesting choroidal vascular hyperpermeability [74]. Although the other study did not compare risk allele frequencies against normal controls, it reported that the risk allele frequencies in patients with tnAMD/PCV manifesting choroidal vascular hyperpermeability were lower than those in patients without choroidal vascular hyperpermeability [78].

In eyes with tnAMD and PCV, the choroidal thickness is reportedly greater in eyes with choroidal vascular hyperpermeability [68,78]. Choroidal vascular hyperpermeability may induce choroidal thickening in eyes with tnAMD/PCV. Consistent with the non-significant genetic associations in eyes with choroidal vascular hyperpermeability, studies from Japan have suggested that the associations of *CFH* and *ARMS2/HTRA1* are weaker in tnAMD/PCV with a thicker choroid than in tnAMD/PCV with a thinner choroid. A study on PCV demonstrated that SFCT was thinner in eyes of patients with risk alleles in *CFH* or *ARMS2/HTRA1*, indicating that the associations of *CFH* and *ARMS2/HTRA1* to PCV were stronger in eyes with a thinner choroid [138]. A study on tnAMD/PCV also supported such an association of *CFH* with choroidal thickness in PCV (*P* = 0.04) and reported a similar tendency, without statistically significance (*P* = 0.12), when tnAMD and PCV were evaluated together [78]. Regarding *ARMS2/HTRA1*, a recent GWAS from Japan has reported that *ARMS2/HTRA1* risk allele causes choroidal thinning in eyes with AMD, resulting in a stronger association of *ARMS2/HTRA1* with AMD in eyes with a thinner choroid [139].

These findings suggest that tnAMD/PCV without choroidal vascular hyperpermeability should be regarded as true and genuine tnAMD/PCV, while tnAMD/PCV with choroidal vascular hyperpermeability should belong to another disease spectrum. It might also be suggested that tnAMD/PCV with a thin choroid should be regarded as true and genuine tnAMD/PCV, while tnAMD/PCV with a thick choroid should belong to another disease spectrum.

## 9. Emergence of the Pachychoroid Spectrum Disease Concept

Recent OCT angiography can clearly detect type 1 CNV secondary to CSC [140,141,142,143], and even spectral domain OCT can recognize CNV when its size and height are sufficient; however, it was difficult to realize that type 1 CNV development was not rare in eyes with CSC until around 2010, when spectral domain OCT became widely used. In 2012, a case series study described detailed characteristics of patients with type 1 CNVs secondary to CSCs [1]. This study analyzed the following two patient groups with type 1 CNV: patients with long-standing CSC who subsequently developed type 1 CNV and patients with type 1 CNV with features suggestive of long-standing CSC who lacked findings of AMD. Although choroidal vascular hyperpermeability was not evaluated in this study, the mean choroidal thickness was reportedly more than 350 µm in both groups. Since the characteristics were quite similar between these two groups, it was suggested that eyes with type 1 CNV with multimodal imaging findings that were more consistent with long-standing CSC than with AMD should be diagnosed as type 1 CNV secondary to CSC and should be differentiated from type 1 CNV secondary to AMD.

The fact that multimodal imaging modalities can diagnose type 1 CNV secondary to CSC without confirming the previous incidence of CSC indicates that fundus changes that are associated with previous CSCs can be detected with multimodal imaging. Previous existence of subretinal fluid can be detected as a descending tract in FA or fundus autofluorescence (FAF). Other characteristics include increased choroidal thickness and/or dilated choroidal vessels in OCT, reduced fundus tessellation in fundus examinations, RPE disruption in FA/FAF, or choroidal vascular hyperpermeability in ICGA. Some of these changes could be detected even before the serous retinal detachment of CSC develops. Eyes with a high risk of developing CSC can manifest these findings, even if they will not develop serous retinal detachment in the future. Indeed, we sometimes encounter eyes with CSC-associated fundus changes that do not present with serous retinal detachment or a history of serous retinal detachment.

In 2013, the concept of pachychoroid pigment epitheliopathy was introduced [2], which was understood as a forme fruste (incomplete form) of CSC. The report described that eyes with pachychoroid pigment epitheliopathy did not have “any history, clinical evidence, or imaging findings consistent with current or antecedent subretinal fluid but otherwise manifested many of the clinical and imaging findings seen in eyes with classic CSC, including a thickened choroid with a characteristic fundus appearance showing reduced fundus tessellation, a range of RPE abnormalities overlying the areas of choroidal thickening, and corresponding FAF changes.”

In the introduction section of this report on pachychoroid pigment epitheliopathy, another new term, “pachychoroid neovasculopathy”, was proposed for the above mentioned “type 1 CNV in CSC masquerading as neovascular AMD” or “type 1 CNV with associated choroidal thickening”. This report speculated that the pachychoroid-driven disease spectrum was comprised of pachychoroid pigment epitheliopathy and CSC, which may progress to pachychoroid neovasculopathy and ultimately to PCV.

**Figure 3:**
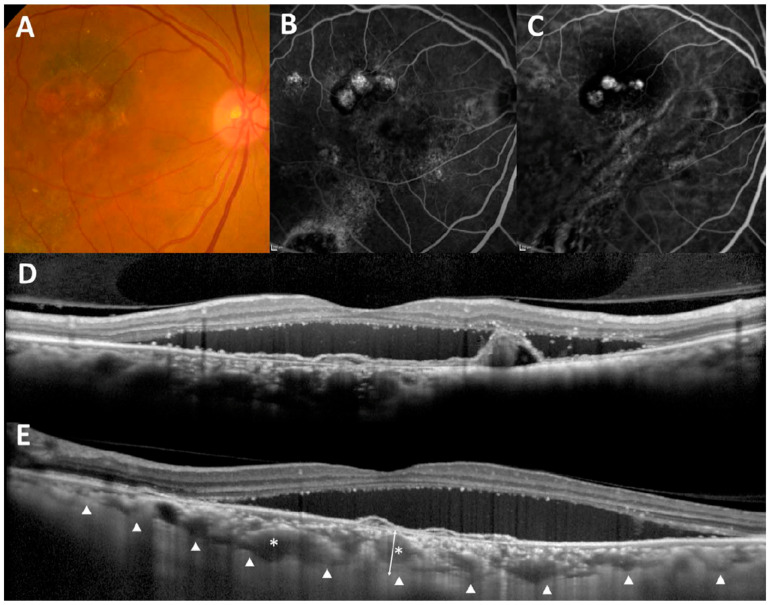
Typical images of pachychoroid neovasculopathy (modified from [149]). Color fundus photography reveals an orange-red nodular lesion, serous retinal detachment, and hard exudates (**A**). Fluorescein angiography reveals a window defect corresponding to RPE atrophy and occult choroidal neovascularization (**B**). Indocyanine green angiography shows choroidal neovascularization and some polypoidal lesions (**C**). A vertical optical coherence tomography (OCT) scan through the fovea shows subretinal fluid and protrusion of retinal pigment epithelium resulting from polypoidal lesions (**D**). A horizontal enhanced depth imaging OCT scan through the fovea shows thickened subfoveal choroidal thickness (triangles) and pachyvessels (asterisks) (**E**).

**Figure 4:**
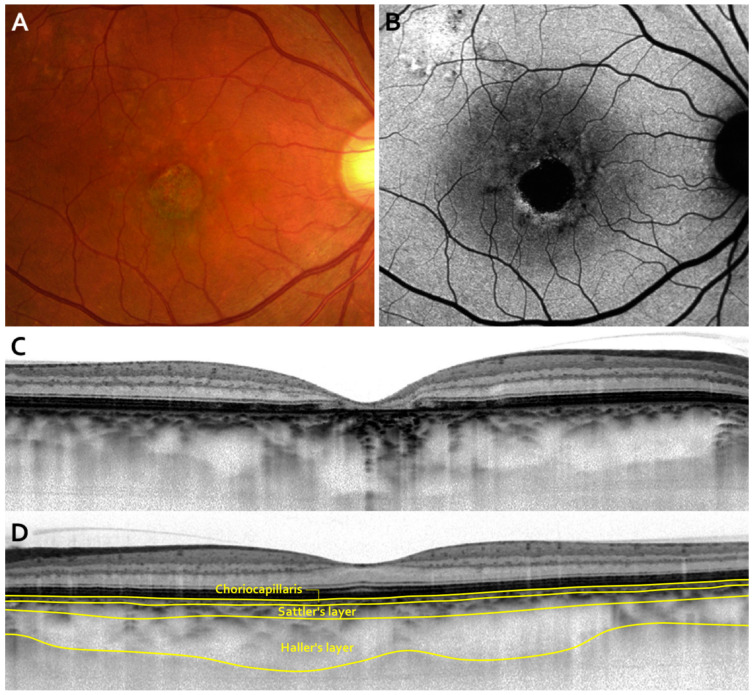
Typical images of pachychoroid geographic atrophy (GA) (modified from [146]). A color fundus photograph shows a reduced fundus tessellation without drusen and central GA (**A**). Fundus autofluorescence images showing hypoautofluorescent areas of GA with surrounding hyper-autofluorescent lesions (**B**). An optical coherence tomography (OCT) image showing markedly dilated large choroidal vessels with obliteration of the choriocapillaris (**C**). The outer nuclear layer is thin and the ellipsoid zone and retinal pigment epithelium bands are disrupted, corresponding to the GA area. An OCT image with thick choroid in the fellow eye. Choriocapillaris layer, Sattler’s layer, and Haller’s layer are indicated (**D**).

Although the above-mentioned report on pachychoroid pigment epitheliopathy did not describe the detailed characteristics of pachychoroid neovasculopathy, a later report from the same group demonstrated three cases of pachychoroid neovasculopathy without a previous history of CSC [3]. According to this report, “eyes with pachychoroid neovasculopathy manifest reduced fundus tessellation on clinical examination and fundus photography, and choroidal thickening and choroidal vascular dilation directly below the neovascular tissue in OCT examination, with obliteration of the choriocapillaris and Sattler’s layer” (Figure 3). It is also reported that “such eyes also show choroidal vascular hyperpermeability in the area surrounding the neovascular tissue in ICGA, RPE abnormalities directly above the dilated choroidal vessels on clinical examination and fundus photography, FAF, and FA without drusen, signs of AMD, myopic degeneration, or other degenerative changes”. The dilated outer choroidal vessel in the Haller’s layer was later named the pachyvessel [144]. Pachyvessels are identified in 96% of eyes with thick choroid PCV and in 100% of eyes with CSC [145]. In addition to CNV, pachychoroid can also cause RPE atrophy and induce GA without drusen, which was recently termed as pachychoroid GA (Figure 4) [146]. Recently, it has been suggested that pachychoroid also induces focal choroidal excavation [147,148].

## 10. Definition of Pachychoroid Neovasculopathy

Although various characteristics of pachychoroid neovasculopathy have been documented in several reports and reviews, the definition of pachychoroid neovasculopathy to distinguish it from neovascular AMD was not proposed until 2015 [4]. In the first study to define pachychoroid neovasculopathy, pachychoroid neovasculopathy was diagnosed if all of the following criteria were met: (1) CNV in either eye; (2) SFCTs ≥ 200 µm in both eyes; (3) no drusen or only non-extensive (total area, ≤ 125 µm circle) hard drusen (≤ 63 µm) in both eyes (AREDS category 1, no AMD); (4) CSC or pachychoroid pigment epitheliopathy characteristics; namely, choroidal vascular hyperpermeability, RPE abnormality independent of CNV lesion, the presence of dilated choroidal vessels or thickening below the type 1 CNV, or a history of CSC.

This definition has been used in later studies to compare pachychoroid neovasculopathy and neovascular AMD [149,150,151]. However, the threshold of 200 µm for SFCT may not always be appropriate because the choroidal thickness varies depending on the patient age, refractive error, and axial length, as pointed out recently [152]. Choroidal thickness also decreases with the progression of CNV-induced degenerative changes. Recent GWAS has shown that *ARMS2/HTRA1* promotes choroidal thinning in eyes with AMD [139]. As choroidal thickening is accompanied by the thinning of the choriocapillaris and Sattler’s vessels that overlie the pachyvessels, these mechanisms may be essential for the development of pachychoroid diseases, and pachychoroid neovasculopathy may be quantitatively defined using choriocapillaris thickness or the vascular diameter of the pachyvessel. However, such a quantitative definition has not been established thus far.

## 11. Significance of Differentiating Pachychoroid Neovasculopathy from tnAMD

Until recently, sufficient pathophysiological or clinically rational explanation had not been provided to confirm the significance of differentiating pachychoroid neovasculopathy from neovascular AMD. A study from Japan reported that VEGF concentrations in aqueous humor were significantly lower in pachychoroid neovasculopathy (63.4 ± standard deviation [SD] of 17.8 pg/mL) than that in neovascular AMD (89.8 ± 45.0 pg/mL) (*P* = 0.035) [149]. However, most reported VEGF concentrations of pachychoroid neovasculopathy fitted into within 1 SD of the VEGF concentration in eyes with neovascular AMD. Considering together that most previous studies reported that VEGF concentration in aqueous humor was between 50 pg/mL to 100 pg/mL in East Asian eyes with tnAMD/PCV (Table 4), the reported concentration of VEGF in pachychoroid neovasculopathy could not strongly suggest that pachychoroid neovasculopathy was a different clinical entity that should be differentiated from neovascular AMD. Although another recent study from Japan also reported significant difference in the VEGF concentration between pachychoroid neovasculopathy and neovascular AMD, the reported concentrations were substantially higher than most previously reported values possibly due to the difference in measurement methods of multiplex bead immunoassay: the median value was 153.8 pg/mL in pachychoroid neovasculopathy while the median value was 203.2 pg/mL in neovascular AMD [153].

There has not been clear difference between neovascular AMD and pachychoroid neovasculopathy also in treatment response and treatment outcome after anti-VEGF injections. Studies on Japanese patients reported that the dry macular rate after one injection of anti-VEGF drug was 56% in pachychoroid neovasculopathy while 50% in neovascular AMD (*P* = 1.0) [153] or 81% in pachychoroid neovasculopathy while 59% in neovascular AMD (*P* = 0.089) [151]. Moreover, the dry macular rate after three loading injections of anti-VEGF drugs was reported to be 91% in pachychoroid neovasculopathy while 84% in neovascular AMD (*P* = 0.51) [4]. Furthermore, two studies on Japanese patients reported that visual acuity improvement after anti-VEGF treatment did not significantly differ between pachychoroid neovasculopathy and neovascular AMD [150,151]. Several studies suggested that pachychoroid neovasculopathy had a better treatment response to anti-VEGF treatment. The retreatment-free periods after the initial three loading treatments were significantly longer for pachychoroid neovasculopathy than for neovascular AMD when treated with a *pro re nata* protocol (*P* = 0.01) [4], and the number of anti-VEGF injections significantly differed (13.2 ± 0.5 in pachychoroid neovasculopathy and 13.8 ± 0.4 in neovascular AMD, *P* < 0.05) during two years of treat-and-extend treatment with an anti-VEGF drug [150]. A study on French patients also reported better response of anti-VEGF treatment for pachychoroid neovasculopathy [168]. However, recent studies on Japanese patients who were non-responders to three monthly injections of anti-VEGF drugs suggested that non-responders included more patients with pachychoroid neovasculopathy [169,170]. A study on Korean patients also reported less response to a 3-monthly anti-VEGF treatment for pachychoroid neovasculopathy [171].

## 12. Genetic Studies on Pachychoroid Diseases

AMD-susceptibility genes were evaluated for their associations with pachychoroid neovasculopathy development in the US and Japan. The US study evaluated 50 patients with neovascular AMD, 50 patients with pachychoroid neovasculopathy, and 50 normal subjects and reported that most risk alleles for AMD in *ARMS2* and *CFH* genes also contributed to the development of pachychoroid neovasculopathy [172]. The risk allele frequencies of *CFH* and *ARMS2/HTRA1* were highest in neovascular AMD, slightly lower in pachychoroid neovasculopathy, and significantly lower in normal controls, which suggests that the genetic characteristics of pachychoroid neovasculopathy stand between AMD and normal controls (Table 5). This study excluded patients who exhibited overlapping features of AMD and pachychoroid disease.

A study from Japan examined 39 patients with pachychoroid neovasculopathy and 161 patients with neovascular AMD by dividing all previously-diagnosed neovascular AMD patients into the two groups [4]. This study also found that the *ARMS2* risk allele frequency was highest in neovascular AMD, slightly lower in pachychoroid neovasculopathy, and significantly lower in normal controls. However, regarding the association of *CFH*, the Japanese study reported that the frequency of the *CFH* risk allele in pachychoroid neovasculopathy was not significantly different from that in normal controls and was significantly lower than in neovascular AMD (Table 6).

## 13. Discovery of Genes Associated with Pachychoroid: Genetic Proof of Concept for Pachychoroid Spectrum Disease

Although previous small-scale genetic studies on AMD-associated genes in pachychoroid neovasculopathy did not reveal the true nature of pachychoroid neovasculopathy [4,172], a recent large-scale GWAS on choroidal thickness clearly elucidated the key difference between pachychoroid and drusen-driven AMD. GWAS using a Japanese community-based cohort discovered that *CFH* strongly affected choroidal thickness (*P* = 2.05 × 10^−10^) [6]. Interestingly, the established *CFH* risk alleles for drusen and AMD were protective against choroidal thickening, while *CFH* risk alleles for thicker choroid were protective against the development of drusen and AMD.

These findings suggest that the *CFH* gene determines the fate of the eyes. Eyes with *CFH* risk alleles for AMD tend to develop drusen and late AMD. However, their choroidal thickness tends to become thinner, and thus, the eyes resist the development of pachychoroid diseases. On the other hand, the choroidal thickness tends to increase in eyes with *CFH* risk alleles for pachychoroid. In these eyes, drusen rarely develop, and thus, drusen-driven late AMD rarely develops. However, eyes with a thicker choroid will develop pachychoroid spectrum diseases, such as CSC, pachychoroid pigment epitheliopathy, pachychoroid GA, or pachychoroid neovasculopathy.

The association of *CFH* to choroidal thickness was later confirmed in the Korean population [173]. Although it has not been clearly confirmed whether *CFH* has a similar effect on eyes in Caucasian populations, a previous study suggested similar *CFH* effects in Caucasians. *CFH* allele frequencies were compared in the US between 50 normal controls and 50 subjects with pachychoroid but not with CNV [172]. The frequency of the risk alleles for AMD did not significantly differ between these two groups, possibly due to the small sample size, but they were slightly lower in subjects with pachychoroid and without CNV. Studies that use larger population-based Caucasian cohorts would possibly find significantly lower *CFH* risk allele frequencies for AMD in subjects with pachychoroid, which indicates that *CFH* has similar effects on choroidal thickness in Caucasian and Asian individuals.

From the viewpoint of the *CFH* gene association, drusen-driven AMD and pachychoroid diseases should belong to opposite disease spectrums. Since the true natures of the diseases are totally different and rather opposite, it should be concluded that pachychoroid neovasculopathy must be differentiated from drusen-driven AMD. As for the association of *CFH* with CSC, *CFH* would affect CSC development not through the common mechanisms of AMD development, but through its effect on the development of pachychoroid.

## 14. Associations of AMD Susceptibility Genes with the Fate of Pachychoroid Diseases

The above Japanese GWAS also confirmed previously reported association of *CFH* to CSC development, where *CFH* risk alleles for drusen and AMD were protective against CSC, but did not show significant association of *ARMS2/HTRA1* to CSC. Another recent study from Japan has discovered that both *CFH* and *ARMS2/HTRA1* determine the fate of eyes after developing CSC [7]. Eyes with *CFH* protective allele against AMD tend to spontaneously resolve without treatment within 3 months after the occurrence of serous retinal detachment secondary to CSC while the serous retinal detachment tends to be prolonged more than 3 months in CSC with *CFH* risk allele for AMD. Furthermore, eyes with the risk alleles for AMD in *CFH* or *ARMS2/HTRA1* tend to develop CNV in the later stage of CSC and progress to pachychoroid neovasculopathy.

## 15. Reviewing Previous Findings from the Viewpoint of Genetic Associations

The discovery that pachychoroid and drusen-driven AMD are genetically opposite spectrums provides us with a novel viewpoint to decode previously reported characteristics of image analyses and genetic characteristics. The following sections explain how to interpret previously reported findings to understand the true nature of pachychoroid diseases and AMD.

## 16. Relationship between Pachychoroid Neovasculopathy and tnAMD from the Viewpoint of *ARMS2/HTRA1*

The previously reported *ARMS2* A69S risk allele frequencies in the Japanese population for AMD and pachychoroid diseases are summarized in Table 7. Although the allele frequencies for AMD and controls were reported in many previous studies, the reported frequencies were uniform, and representative studies are shown in the table. The effect of *ARMS2* gene on the development of neovascular AMD can be represented by the difference/ratio of the risk allele frequencies between neovascular AMD (0.57 or 0.60) and controls (0.39 or 0.37), while the effect of *ARMS2* gene on the development of pachychoroid neovasculopathy can be represented by the difference/ratio of the risk allele frequencies between pachychoroid neovasculopathy (0.51) and controls (0.39 or 0.37). *ARMS2/HTRA1* would promote CNV development secondary to pachychoroid with a weaker magnitude than its influence on CNV development in drusen-driven neovascular AMD. Further genetic studies on pachychoroid diseases would help us understand precise pathogenesis of pachychoroid diseases. Since the diagnosis criteria of pachychoroid diseases using imaging modalities have not been established, previous genetic studies on pachychoroid diseases would have to be re-evaluated after the elucidation of their precise pathogenesis.

The effect of *ARMS2/HTRA1* on the development of pachychoroid neovasculopathy can also be reflected on the difference/ratio of the risk allele frequencies between CSC without CNV and CSC with CNV because some of pachychoroid neovasculopathy progresses from CSC by developing type 1 CNV, while the others progress from pachychoroid pigment epitheliopathy. When eyes with CSC were divided into two groups depending on the development of type 1 CNV during the follow-up period, the reported risk allele frequencies of *ARMS2* A69S were 0.32 for CSC without CNV development and 0.54 for CSC with CNV development, while 0.34 for all CSC cases. The higher risk allele frequency in CSC with CNV indicates that *ARMS2* substantially contributes to the pathway of developing pachychoroid neovasculopathy from CSC. So far, genetic characteristics of pachychoroid pigment epitheliopathy have not been clearly elucidated. Elucidation of genetic characteristics of pachychoroid pigment epitheliopathy would further lead to a precise understanding of the nature of pachychoroid diseases.

Previously reported characteristics of *ARMS2* on choroidal vascular hyperpermeability in AMD further confirm that choroidal vascular hyperpermeability is one of the most important characteristics of pachychoroid neovasculopathy. The risk allele frequencies of pachychoroid neovasculopathy (0.51) and CSC with CNV development (0.54) are relatively lower than those of AMD without choroidal vascular hyperpermeability (0.67 or 0.60) and rather near to those of AMD with choroidal vascular hyperpermeability (0.58, 0.42, or 0.40). Pachychoroid neovasculopathy would be nearer to AMD with choroidal vascular hyperpermeability than AMD without choroidal vascular hyperpermeability. The *ARMS2* viewpoint confirms that it is reasonable to differentiate pachychoroid neovasculopathy from tnAMD by focusing on choroidal vascular hyperpermeability.

## 17. Relationship between Pachychoroid Neovasculopathy and tnAMD from the Viewpoint of *CFH*

Previously reported *CFH* I62V risk allele frequencies in Japanese for AMD and pachychoroid diseases are summarized in Table 8. The effect of the *CFH* gene on the development of neovascular AMD can be represented by the difference/ratio of the risk allele frequencies between neovascular AMD (0.75 or 0.73) and controls (0.59). As *CFH* substantially affect choroidal thickness before the development of diseases, the effect of the *CFH* gene on the development of pachychoroid neovasculopathy from the pachychoroid status should be evaluated by using the difference/ratio of the risk allele frequencies between pachychoroid neovasculopathy and its background of pachychoroid without CNV, not between pachychoroid neovasculopathy and control with all range of choroidal thickness. The reported risk allele frequencies of *CFH* I62V in pachychoroid neovasculopathy was 0.59, while the risk allele frequencies were 0.59 in control with SFCT 250–350 µm, 0.58 in control with SFCT 350–450 µm, 0.50 in control with SFCT 450–550 µm, and 0.46 in controls with SFCT greater than 550 µm. These risk allele frequencies indicate that the *CFH* risk allele for AMD also contributes to the development of pachychoroid neovasculopathy.

The risk allele frequencies in CSC with and without CNV also suggest that *CFH* is associated with the development of pachychoroid neovasculopathy. When eyes with CSC were divided into two groups depending on the development of type 1 CNV during the follow-up period, the reported risk allele frequencies of *CFH* I62V were 0.50 for CSC without CNV development and 0.79 for CSC with CNV development, while it was 0.52 for all CSCs.

In contrast to *ARMS2/HTRA1*, *CFH* risk allele frequencies cannot simply reflect the importance of choroidal vascular hyperpermeability in pachychoroid neovasculopathy possibly because *CFH* significantly affects choroidal thickness before development of pachychoroid neovasculopathy or AMD. Considering that a thick choroid and choroidal vascular hyperpermeability often coexist, *CFH* potentially has two-edged roles of promoting choroidal thickening/choroidal vascular hyperpermeability and CNV development both in drusen-driven AMD and pachychoroid neovasculopathy (Figure 1C).

Regarding CNV secondary to high myopia, both *CFH* and *ARMS2/HTRA1* did not affect its development [174,175,176]. *CFH* and *ARMS2/HTRA1* are not always associated with CNV development, and CNV development secondary to pachychoroid and CNV development in drusen-driven neovascular AMD would share pathophysiological mechanisms to some extent. In mice, increased expression of HTRA1 in retinal pigment epithelium reportedly induced PCV [177]. Further basic research from the genetic view point of AMD and pachychoroid diseases would be warranted.

## 18. Relationship between Pachychoroid Neovasculopathy and CSC

In 1992, a study from Japan reported that a history of CSC was a risk factor for developing neovascular AMD [178]. As the concept of PCV was not yet widely accepted around 1992, this study did not classify neovascular AMD into tnAMD and PCV. After the introduction of PCV, many Japanese ophthalmologists evaluated CSC history in patients with tnAMD and PCV [64,65,179,180]. One study reported more instances of CSC history in eyes with PCV than in eyes with tnAMD and suggested that CSC history was a risk factor for PCV [179]. However, later studies reported that CSC characteristics were observed similarly in tnAMD and PCV. Choroidal vascular hyperpermeability was observed in 30–50% of tnAMD and PCV, and choroidal thicknesses in eyes with tnAMD/PCV manifesting choroidal vascular hyperpermeability was similar to those in eyes with CSC in patients of similar ages [46,53,65,66,67,68,69,70,71,72,73,78]. Realizing the difficulty in differentiating tnAMD, PCV, and CSC by studying the CSC characteristics in tnAMD/PCV, many Japanese retina specialists have speculated the existence of a common pathogenic mechanism between tnAMD/PCV and CSC [64,65,181].

After the genetic confirmation of the pachychoroid disease concept, the previously suggested common pathogenic mechanism between tnAMD/PCV and CSC was found to be pachychoroid. As explained above, genetic characteristics of tnAMD/PCV suggest that some of the previously diagnosed tnAMD/PCV developed from a pachychoroid background and the rest of the previously diagnosed tnAMD/PCV belong to drusen-driven AMD. Most previously diagnosed tnAMD/PCV with a thicker choroid and choroidal vascular hyperpermeability should develop from the background of pachychoroid while most tnAMD/PCV with a thinner choroid and less choroidal vascular hyperpermeability should develop through drusen-driven mechanisms.

## 19. Relationship between Pachychoroid Neovasculopathy and PCV

The viewpoint of *ARMS2* helps us realize that the two previously proposed subtypes of PCV had also suggested the existence of pachychoroid neovasculopathy. Two Japanese groups and a French group divided PCV into “smaller PCV” and “larger PCV”, typical PCV” and “polypoidal CNV”, or “idiopathic PCV” and “PCV as a subtype of neovascular AMD” (Table 1). The “smaller PCV”, typical PCV”, and “idiopathic PCV” share similar clinical and genetic characteristics while “larger PCV”, “polypoidal CNV”, and “PCV as a subtype of neovascular AMD” share similar clinical and genetic characteristics. The relatively lower *ARMS2* risk allele frequencies in the former subtypes suggest that most eyes of “smaller PCV”, “typical PCV,” and “idiopathic PCV” could belong to pachychoroid neovasculopathy, while the relatively higher *ARMS2* risk allele frequencies in the later subtypes suggest that most eyes with “larger PCV”, “polypoidal CNV”, and “PCV as a subtype of neovascular AMD” could belong to drusen-driven AMD. Although some previous studies considered that PCV belongs to pachychoroid spectrum disease [182,183], a subset of PCVs should belong to drusen-driven neovascular AMD rather than to the pachychoroid spectrum disease. Recently, a classification system for pachychoroid diseases was proposed: 0, uncomplicated pachychoroid; I, pachychoroid pigment epitheliopathy; II, CSC; III, pachychoroid neovasculopathy; and IV, pachychoroid aneurysmal type 1 CNV (formerly PCV). [184] Although this classification system is reasonable, we should not forget that a subset of PCVs does not belong to pachychoroid diseases.

Although the first study on pachychoroid neovasculopathy reported that pachychoroid neovasculopathy is often accompanied with polypoidal lesions [1] and the later case report of three patients with pachychoroid neovasculopathy also speculated that pachychoroid neovasculopathy could ultimately progress to PCV [3], yet another later study from the same group in the US reported that the prevalence of polypoidal lesions was only 18% in patients with pachychoroid neovasculopathy [144]. A study in Japan demonstrated that the prevalence of polypoidal lesions did not significantly differ between pachychoroid neovasculopathy (56%) and neovascular AMD (43%) [4]. Other studies from Japan reported that 33% or 38% of pachychoroid neovasculopathies were accompanied by polypoidal lesions [150,151]. It is suggested that some eyes with pacychoroid neovasculopathy do not develop polypoidal lesions and maintain their type 1 CNV without polypoidal lesion forever, and not all cases of pachychoroid neovasculopathy progress to PCV.

## 20. Subtypes of Neovascular AMD Rearranged from Genetic Viewpoints

**Figure 5:**
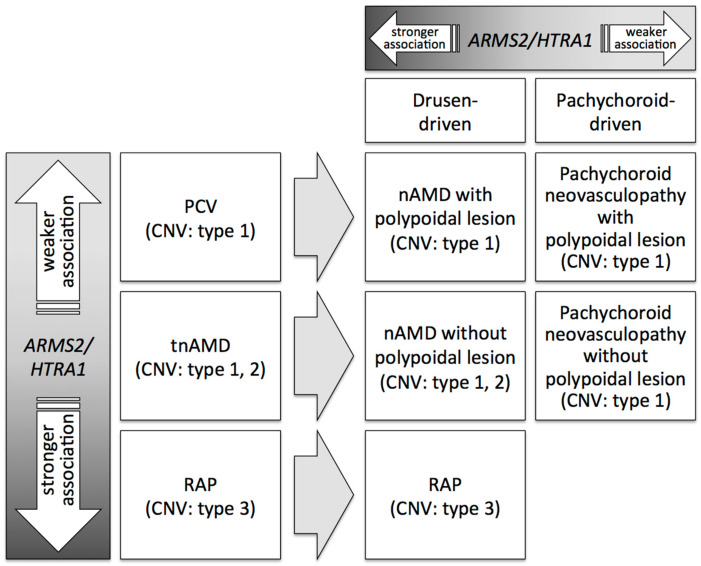
Associations of *ARMS2/HTRA1* with subtypes of age-related macular degeneration (AMD) before and after introduction of the pachychoroid concept. Before the pachychoroid concept, AMD was divided into three subtypes of polypoidal choroidal vasculopathy (PCV), typical neovascular AMD (tnAMD), and retinal angiomatous proliferation (RAP). The association of *ARMS2/HTRA1* is weaker for PCV and stronger for RAP than tnAMD. After the pachychoroid concept, PCV is further divided into the drusen-driven type and pachychoroid-driven type, and tnAMD is also divided into the drusen-driven type and pachychoroid-driven type.

In 2012, it was proposed that some polypoidal lesions of PCV should be understood as the deformation of the edge of type 1 CNV [124]. Furthermore, a recent report proposed that polypoidal lesions of PCV should be understood as an aneurysmal change at the edge of type 1 CNV secondary to various backgrounds [185]. It might be better to withhold using the disease name of PCV and simply to describe CNV by adding the terms “with polypoidal lesion” or “without polypoidal lesion”. As CNV can develop by both a drusen-driven mechanism and a pachychoroid-driven mechanism, previously diagnosed tnAMD/PCV can be classified into drusen-driven neovascular AMD with polypoidal lesion, drusen-driven neovascular AMD without polypoidal lesion, pachychoroid neovasculopathy with polypoidal lesion, and pachychoroid neovasculopathy without polypoidal lesion (Figure 5).

Among cases of neovascular AMD diagnosed before the pachychoroid concept era, the association of *ARMS2/HTRA1* was reportedly stronger in RAP and weaker in PCV. [83,116,119,120]. This can be partly explained by the relatively weaker association of *ARMS2/HTRA1* with pachychoroid neovasculopathy. As RAP does not include pachychoroid neovasculopathy, and PCV and tnAMD include pachychoroid neovasculopathy, the association of *ARMS2/HTRA1* becomes stronger in RAP than in PCV and tnAMD. Regarding the difference between PCV and tnAMD, the existence of type 2 CNV can further explain the difference in the association. The CNV in pachychoroid neovasculopathy is usually a type 1 CNV, which suggests that eyes with type 2 CNV is drusen-driven AMD and the association of *ARMS2/HTRA1* is stronger in type 2 CNV. As tnAMD includes eyes with type 2 CNV while PCV does not, the association of *ARMS2/HTRA1* becomes stronger in tnAMD than in PCV. Since the difference in the clinical characteristics between PCV and tnAMD is not sharply separated from the multimodal imaging as discussed above, previous genetic studies on PCV and tnAMD should be carefully evaluated paying attention to the ambiguity of their diagnosis. It might be better to investigate genetic characteristics of eyes with polyps by dividing neovascular AMD into drusen-driven type and pachychoroid-driven type.

**Figure 6:**
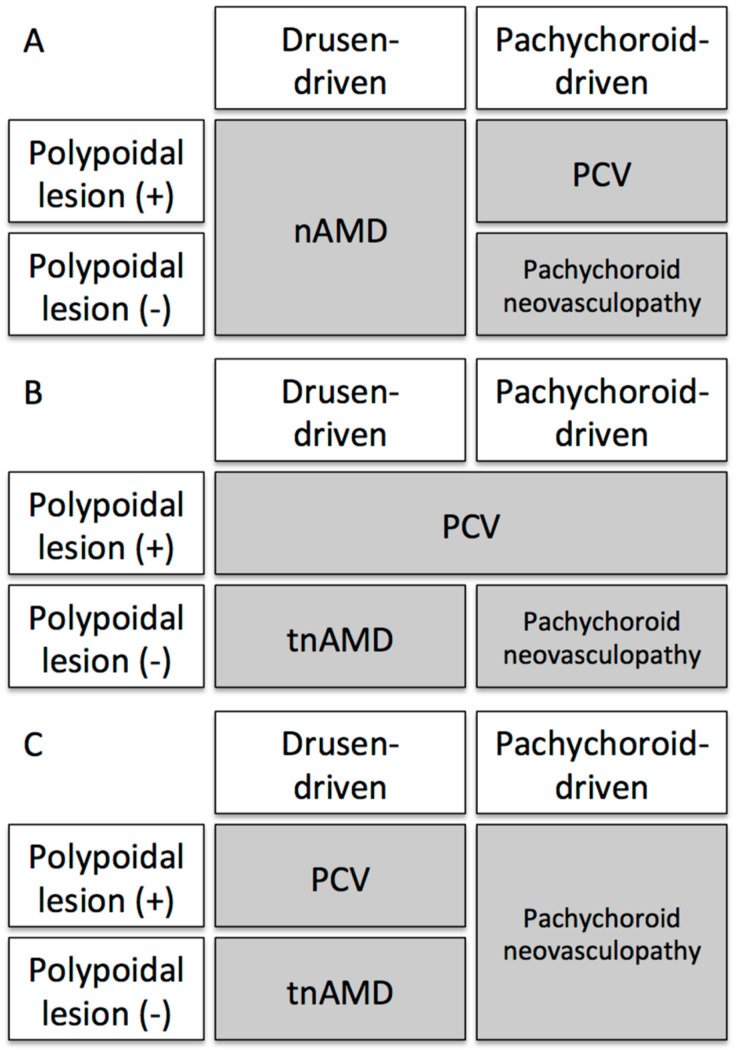
The range of polypoidal choroidal vasculopathy (PCV) and pachychoroid neovasculopathy covers to be discussed. When the usage of PCV is limited to “pachychoroid driven disease with polypoidal lesion”, pachychoroid neovasculopathy should cover the range of “pachychoroid-driven disease with choroidal neovascularization (CNV) but without polypoidal lesion” (**A**). When both “drusen-driven CNV with polypoidal lesion” and “pachychoroid-driven CNV with polypoidal lesion” into PCV, then “pachychoroid neovasculopathy without polypoidal lesion” should be true pachychoroid neovasculopathy and “drusen-driven neovascular AMD without polypoidal lesion” might have to be called “drusen-driven typical neovascular AMD” (**B**). When we apply pachychoroid neovasculopathy regardless of the existence of a polypoidal lesion, “drusen-driven neovascular AMD with polypoidal lesion” might be called PCV (**C**).

Thus far, previous studies have not compared the genetic characteristics between drusen-driven neovascular AMD with a polypoidal lesion and drusen-driven neovascular AMD without a polypoidal lesion. The genetic differences between pachychoroid neovasculopathy with a polypoidal lesion and pachychoroid neovasculopathy without a polypoidal lesion have not also been investigated. If future studies reveal that the genetic background differs between drusen-driven neovascular AMD with polypoidal lesion and drusen-driven neovascular AMD without polypoidal lesion or between pachychoroid neovasculopathy with polypoidal lesion and pachychoroid neovasculopathy without polypoidal lesion, we should continue using the term of PCV. Furthermore, if definitive differences are found in the clinical course or treatment outcome between drusen-driven neovascular AMD with polypoidal lesion and drusen-driven neovascular AMD without polypoidal lesion or pachychoroid neovasculopathy with polypoidal lesion and pachychoroid neovasculopathy without polypoidal lesion, we should continue using the term of PCV. To continue using the term of PCV, subtype(s) included under PCV and pachychoroid neovasculopathy should be further discussed (Figure 6).

Previous studies from two study groups suggested that “pachychoroid neovasculopathy with polypoidal lesion” is a true PCV (Figure 6A) [122,123,124,125]. If so, “pachychoroid neovasculopathy without polypoidal lesion” should be considered as true pachychoroid neovasculopathy, and PCV and pachychoroid neovasculopathy can be clearly separated from drusen-driven neovascular AMD. In contrast, many ophthalmologists had included both “drusen-driven neovascular AMD with polypoidal lesion” and “pachychoroid neovasculopathy with polypoidal lesion” as PCV (Figure 6B). With this archaic usage of PCV, “pachychoroid neovasculopathy without polypoidal lesion” should be considered as true pachychoroid neovasculopathy and “drusen-driven neovascular AMD without polypoidal lesion” might have to be called “drusen-driven typical neovascular AMD”. If we apply pachychoroid neovasculopathy regardless of the existence of polypoidal lesion, “drusen-driven neovascular AMD with polypoidal lesion” might be called PCV (Figure 6C).

## 21. Personalized/Precision Medicine for CSC

Recently, it has been demonstrated that the genotype information of *CFH* and *ARMS2/HTRA1* can predict the spontaneous resolution of CSC and progression from CSC to pachychoroid neovasculopathy [7]. CSC in patients with *CFH* protective alleles for AMD tend to resolve spontaneously within 3 months, while CSC in patients with *CFH* risk alleles for AMD tend not to resolve spontaneously within 3 months. When we treat patients with CSC, we might be better to avoid early treatment for patients with the protective alleles while we should consider early treatment for patients with the risk alleles. Furthermore, CSC with risk alleles for AMD in *CFH* or *ARMS2/HTRA1* tends to progress to pachychoroid neovasculopathy by developing CNV while CSC with protective alleles for AMD does not. We might be able to prevent or delay the progression from CSC to pachychoroid neovasculopathy or minimize the CNV lesion size in pachychoroid neovasculopathy by performing early treatment for CSC in patients with the risk alleles for pachychoroid neovasculopathy. Prospective studies should confirm the effects of early treatment for CSC on the progression of pachychoroid neovasculopathy by paying attention to the genetic risk for pachychoroid neovasculopathy in each patient. Personalized/precision medicine for CSC would be able to attain better long-term visual prognosis for patients with CSC.

Genetic associations with clinical course and treatment outcome for AMD have been eagerly investigated, although prospective study has been performed rarely [186]. Many studies have suggested that *ARMS2/HTRA1* polymorphisms can predict neovascular AMD bilaterality or fellow eye development [187,188,189,190,191,192], although this has not been fully confirmed [193]. In PCV, *ARMS2/HTRA1* might be able to predict the occurrence of subretinal or vitreous hemorrhages [189,194]. However, with respect to genetic associations with treatment outcomes, a useful gene has not been discovered for personalized/precision medicine for neovascular AMD. Previous failure of discovering genes associated with treatment outcome might be due to its study design including both drusen-driven AMD and pachychoroid neovasculopathy into one disease entity of AMD. Since drusen-driven AMD and pachychoroid neovasculopathy have genetically opposite background, genes associated with treatment outcome should be investigated in drusen-driven AMD and pachychoroid neovasculopathy, separately.

## 22. Future Research

Drusen are usually observed in elderly people and were classified into hard drusen and soft drusen. In 2010, however, “drusen-like lesion” was reported to be found in 21 eyes (51%) of 41 eyes with active CSC in Japanese patients <50 years of age [195]. Furthermore, the concept of pachydrusen has recently been proposed [152]. According to the report, pachydrusen develops in eyes with thick choroids and are typically larger than 125 mm, often have irregular outer contour, show a scattered distribution over the posterior pole, and occur in isolation or in groups of only a few drusen.

Roles of pachydrusen in the development of pachychoroid diseases have not been elucidated. Although pachyvessels are demonstrated to locate under pachydrusen both in AMD and CSC [196,197], it has not been concluded whether pachydrusen causes pachyvessels or vice versa. The genetic background of pachydrusen has also not been fully elucidated. Only one group evaluated risk alleles for AMD in *CFH* and *ARMS2* and reported that risk alleles in *CFH* and *ARMS2* were less frequent in eyes with pachydrusen than eyes with soft drusen (*P* = 0.0091 and 2.4 × 10^−8^, respectively), and the risk allele in *CFH* was significantly less frequent in eyes with pachydrusen than in eyes without drusen (*P* = 0.011) [198]. To discuss the relationships of pachydrusen, pachychoroid diseases, and drusen-driven AMD, genetic studies have to elucidate background of pachydrusen further. Since pachydrusen are often observed in Asian eyes with tnAMD or PCV [199,200,201], genetic characteristics and clinical significance of pachychoroid should be intensively investigated in Asian countries.

The vague border between pachychoroid neovasculopathy and drusen-driven neovascular AMD is another issue for the future. The first report to establish the definition of pachychoroid neovasculopathy and differentiate this disease from neovascular AMD included, as a criterion, that subfoveal choroidal thickness should be ≥ 200 µm in both eyes [4]. However, in the first case report of three patients with pachychoroid neovasculopathy, the subfoveal choroidal thickness of the fellow eyes were 155, 210, and 150 µm [3]. Since choroidal thickness changes with age and during the progression of macular diseases, pachyvessels and thinning of choriocapillaris and Sattler’s vessels overlying the pachyvessels provide more practical characteristics regarding pachychoroid spectrum diseases. The definition of pachychoroid neovasculopathy should focus on the presence of pachyvessels and the thinning of choriocapillaris, rather than on choroidal thickness. Recently, the presence of an asymmetric vortex vein has been reported as a new characteristic of CSC [202] and asymmetry of choroidal vessels in Haller’s layer can be automatically evaluated with en face OCT images [203]. An algorithm can be potentially installed into OCT or OCT angiography machines to quantitatively evaluate choroidal vessels and automatically diagnose the pachychoroid status.

Some previous reports have abbreviated pachychoroid neovasculopathy as PNV. However, NV usually stands for neovascularization. As neovascularization and neovasculopathy are different entities, the use of NV for neovasculopathy is not appropriate. PCN or PN might be a better abbreviation for pachychoroid neovasculopathy.

## 23. Conclusions

As the concept of pachychoroid diseases was proposed through clinically acknowledged characteristics of image examination, objective evidence to prove this concept was anticipated. Our recent GWAS has discovered that pachychoroid and drusen-driven AMD belong to opposite spectrums with regard to the *CFH* gene. This novel genetic viewpoint supports and strengthens the concept of pachychoroid disease as a clinical entity distinct from drusen-driven AMD. Once we understand the genetic background of pachychoroid diseases, we can realize that previous genetic studies already supported the concept of pachychoroid diseases. The similarity of choroidal thickness between CSC and tnAMD/PCV with choroidal vascular hyperpermeability and the non-significant associations of *CFH* to tnAMD/PCV with choroidal vascular hyperpermeability in previous reports indicate that tnAMD/PCV with choroidal vascular hyperpermeability and a thick choroid is mostly pachychoroid neovasculopathy rather than drusen-driven neovascular AMD. Previously proposed characteristics of pachychoroid neovasculopathy, such as frequent choroidal vascular hyperpermeability and a thick choroid are now confirmed by objective evidence of genetic associations. Furthermore, a detailed genetic analysis of PCV together with detailed image analyses revealed that PCV should be divided into the following two groups: pachychoroid-driven CNV with polypoidal lesion and drusen-driven CNV with polypoidal lesion. Similarly, tnAMD should be divided into pachychoroid neovasculopathy without polypoidal lesions and drusen-driven neovascular AMD without polypoidal lesions. Future studies should investigate clinical, genetic, and image analysis characteristics to understand the true nature of AMD and pachychoroid diseases and improve treatment outcomes for these diseases.

## Figures and Tables

**Table 1:** Characteristics of two subtypes of polypoidal choroidal vasculopathy.

	Tsujikawa A et al. [121]	Tanaka, K et al. [122] Yuzawa M et al. [123,124]	Coscas G et al. [125]
	Larger PCV	Polypoidal CNV (Deformation of CNV at its edge)	Neovascular AMD-related PCV
Greatest linear dimension (µm)	3915 ± 1591	4759 ± 1655	3292 ± 1542
Area of lesion (mm^2^)	9.79 ± 10.55	-	-
Choroidal thickness (µm)	-	-	177 ± 63
Drusen detected	-	-	100%
*ARMS2/HTRA1* A69S T allele frequency	61%	76%	-
	Smaller PCV	Typical PCV PCV in the narrow and strict sense (Abnormalities of the choroidal vessels)	Idiopathic PCV
Greatest linear dimension (µm)	1991 ± 464	1798 ± 738	1648 ± 913
Area of lesion (mm^2^)	1.68 ± 0.53	-	-
Choroidal thickness (µm)	-	-	278 ± 100
Drusen detected	-	-	15%
ARMS2/HTRA1 A69S T allele frequency	40%	39%	-

PCV, polypoidal choroidal vasculopathy; CNV, choroidal neovascularization; AMD, age-related macular degeneration.

**Table 2:** Risk allele frequencies of *CFH* I62V in Japanese patients with age-related macular degeneration.

	CVH (+)	CVH (-)	Control	
PCV	63% (*N* = 35)	74% (*N* = 114)	60%	Yoneyama [138]
tnAMD/PCV with type 1 CNV	61% (*N* = 42)	-	58%, 61%	Miyake [74]
tnAMD/PCV	66% (*N* = 31)	76% (*N* = 54)	-	Jirarattanasopa [78]

CVH, choroidal vascular hyperpermeability.

**Table 3:** Risk allele frequencies of *ARMS2* A69S in Japanese patients with age-related macular degeneration.

	CVH (+)	CVH (-)	Control	
PCV	40% (*N* = 35)	60% (*N* = 114)	38%	Yoneyama [138]
tnAMD/PCV with type 1 CNV	42% (*N* = 42)	-	37%/43%	Miyake [74]
tnAMD/PCV	58% (*N* = 32)	67% (*N* = 54)	-	Jirarattanasopa [78]

CVH, choroidal vascular hyperpermeability.

**Table 4:** Previously reported VEGF concentration (pg/mL) in aqueous humor of eyes with age-related macular degeneration.

	Area	Subtype	N	Mean	SD	SEM	95%PI	Median	IQR	Measurement Method
Hu et al. [154]	China	Refractory PCV *	41	55	27	-	-	52	-	Immunoassay using multi-analyte biochip array (RANDOX laboratories)
		Stable PCV *	39	43	23	-	-	33	-	
Sasaki et al. [155]	Japan	PCV	62	55	-	5	-	59	23-84	ELISA using Q-Plex kit (Quansys Biosciences)
Nomura et al. [156]	Japan	tnAMD/PCV with PVD	13	58	-	-	25-137	-	-	ELISA using Quantikine Kit (R&D Systems)
		tnAMD/PCV without PVD	20	91	-	-	31-267	-	-	
Lee MY et al. [157]	Korea	PCV	21	62	-	37	-	-	-	Multiplex bead immunoassay using LINCOplex kit (LINCO Research)
Kim JH et al. [158]	Korea	nAMD	30	63	32	-	-	-	-	ELISA using human VEGF immunoassay (Thermo Fischer Scientific)
Agawa T et al. [159]	Japan	tnAMD/PCV	37	67	43	-	-	-	-	Flow cytometry using Cytometric Bead Array (PharMingen)
Roh MI et al. [160]	Korea	Naïve nAMD	5	67	35	-	-	-	-	Multiplex biochip immunoassay using Cytokine Array (RANDOX laboratories)
		Recurrent nAMD	14	56	63	-	-	-	-	
Roh MI et al. [161]	Korea	nAMD	10	68	32	-	-	-	-	Immunoassay using multi-analyte biochip array (RANDOX laboratories)
Sakurada Y et al. [162]	Japan	PCV	22	69	27	-	-	-	-	ELISA using Q-Plex kit (Quansys Biosciences)
		tnAMD	18	71	16	-	-	-	-	
Wang X et al. [163]	Japan	tnAMD/PCV with monthly ranibizumab treatment	9	95	32	-	-	-	-	ELISA using Quantikine Kit (R&D Systems)
		tnAMD/PCV with bimonthly ranibizumab treatment	17	152	80	-	-	-	-	
Chan WM et al. [164]	Hong Kong	tnAMD	34	103	91	-	-	-	-	ELISA using ChemiKine kit (Chemicon International)
Cha DM et al. [165]	Korea	tnAMD	20	122	63	-	-	-	-	RayBio Biotin Label-Based Cytokine Antibody Array (RayBiotech)
Sato T et al. [166]	Japan	tnAMD/PCV	21	228	176	-	-	-	-	Multiplex bead immunoassay using Bio-Plex kit (Bio-rad)
Tong JP et al. [167]	Hong Kong	PCV	11	403	230	-	-	-	-	ELISA using ChemiKine kit (Chemicon International)
		tnAMD	12	669	340	-	-	-	-	

SD, standard deviation; SEM, standard error of the mean; PI, prediction interval; IQR, interquartile range; PCV, polypoidal choroidal vasclopathy; tnAMD, typical neovascular age-related macular degeneration; nAMD, neovascular age-related macular degeneration; ELISA, enzyme-linked immunosorbent assay; PVD, posterior vitreous detachment. * Refractory PCV patients were defined as those showing signs of recurrence or persistence of the lesions at 12 months follow-up after the latest treatment/re-treatment with anti-VEGF monotherapy or combination of photodynamic therapy and anti VEGF treatment, while stable PCV patients were defined as those whose polyps completely regressed at 12 months follow-up.

**Table 5:** Risk allele frequencies for age-related macular degeneration in Caucasians.

Dansingani KK et al. [172]	*ARMS2*	*CFH*		
	A69S-T	Y402H-C	rs2274700-C	rs12144939-G
AMD (*n* = 50)	0.44	0.63	0.82	0.87
Pachychoroid with neovascularization (*n* = 50)	0.41	0.46	0.64	0.84
Control (*n* = 50)	0.22	0.31	0.48	0.73
Pachychoroid without neovascularization (*n* = 50)	0.15	0.24	0.39	0.64

AMD, age-related macular degeneration.

**Table 6:** Risk allele frequencies for age-related macular degeneration in Japanese.

	*ARMS2* A69S-T		*CFH* I62V-G	
AMD	0.60	(*N* = 1336) [83]	0.75	(*N* = 1338) [83]
	0.57	(*N* = 1536) [107]	0.73	(*N* = 1536) [107]
Pachychoroid neovasculopathy	0.51	(*N* = 39) [4]	0.59	(*N* = 39) [4]
Control	0.39	(*N* = 950) [83]	0.59	(*N* = 947) [83]
	0.37	(*N* = 3246) [4]	0.59	(*N* = 3246) [4]

AMD, age-related macular degeneration.

**Table 7:** Previously reported risk allele frequencies of *ARMS2* A69S in Japanese.

	RAF		
	0.90		RAP [83]
	.		
	0.70		
	0.69		
	0.68		
tnAMD/PCV without CVH [78]	0.67		
	0.66		
	0.65		
	0.64		tnAMD [83]
	0.63		
	0.62		
	0.61		
PCV without CVH [138]	0.60	AMD [83]	
	0.59		
tnAMD/PCV with CVH [78]	0.58		
	0.57	AMD [107]	
	0.56		
	0.55		PCV [83]
	0.54		CSC with CNV development [7]
	0.53		
	0.52		
Pachychoroid neovasculopathy [4]	0.51		
	0.50		
	0.49		
	0.48		
	0.47		
	0.46		
	0.45		
	0.43		
Type 1 CNV with CVH [74]	0.42		
	0.41		
PCV with CVH [138]	0.40		
	0.39	Control [83]	
	0.38		
	0.37	Control [4]	
	0.36		
	0.35		
	0.34	CSC [7]	
	0.33		
	0.32		CSC without CNV development [7]

RAF, risk allele frequency; CSC, central serous chorioretinopathy; RAP, retinal antiomatous proliferation; tnAMD, typical neovascular age-related macular degeneration; PCV, polypoidal choroidal vasculopathy; CVH, choroidal vascular hyperpermeability; AMD, age-related macular degeneration; CNV, choroidal neovascularization.

**Table 8:** Previously reported risk allele frequencies of *CFH* I62V in Japanese.

	RAF		
	0.79		CSC with CNV [7]
	0.78		
	0.77		
tnAMD/PCV without CVH [78]	0.76		
	0.75	AMD [83]	
PCV without CVH [138]	0.74		
	0.73	AMD [107]	
	0.72		
	0.71		
	0.70		
	0.69		
	0.68		
	0.67		
tnAMD/PCV with CVH [78]	0.66		Control (SFCT: 50–150 µm) [6]
	0.65		
	0.64		
PCV with CVH [138]	0.63		
	0.62		Control (SFCT: 150–250 µm) [6]
Type 1 CNV with CVH [74]	0.61		
	0.60		
pachychoroid neovasculopathy [4]	0.59	Control [4,83]	Control (SFCT: 250–350 µm) [6]
	0.58		Control (SFCT: 350–450 µm) [6]
	0.57		
	0.56		
	0.55		
	0.54		
	0.53		
	0.52	CSC [7]	
	0.51		
	0.50		Control (SFCT:450–550 µm) [6] CSC without CNV [7]
	0.49		
	0.48		
	0.47	CSC [127]	
	0.46	CSC [6]	Control (SFCT:550 µm-) [6]

RAF, risk allele frequency; CSC, central serous chorioretinopathy; CNV, choroidal neovascularization; tnAMD, typical neovascular age-related macular degeneration; PCV, polypoidal choroidal vasculopathy; CVH, choroidal vascular hyperpermeability; AMD, age-related macular degeneration; SFCT, subfoveal choroidal thickness.
